# Predictors of intensive care unit admission in patients with *Legionella* pneumonia: role of the time to appropriate antibiotic therapy

**DOI:** 10.1007/s15010-020-01565-7

**Published:** 2020-12-14

**Authors:** Marco Falcone, Alessandro Russo, Giusy Tiseo, Mario Cesaretti, Fabio Guarracino, Francesco Menichetti

**Affiliations:** 1 Division of Infectious Diseases, Department of Clinical and Experimental Medicine, University of Pisa, Pisa, Italy; 2 Department of Anaesthesia and Critical Care, Cardiothoracic and Vascular Anaesthesia and Intensive Care Medicine, Azienda Ospedaliero Universitaria Pisana, Pisa, Italy

**Keywords:** *Legionella*, Intensive care unit, Pneumonia, Cardiovascular events, Empirical antibiotic therapy

## Abstract

**Purpose:**

*Legionella* spp. pneumonia (LP) is a cause of community-acquired pneumonia (CAP) that requires early intervention. The median mortality rate varies from 4 to 11%, but it is higher in patients admitted to intensive care unit (ICU). The objective of this study is to identify predictors of ICU admission in patients with LP.

**Methods:**

A single-center, retrospective, observational study conducted in an academic tertiary-care hospital in Pisa, Italy. Adult patients with LP consecutively admitted to study center from October 2012 to October 2019.

**Results:**

During the study period, 116 cases of LP were observed. The rate of ICU admission was 20.7% and the overall 30-day mortality rate was 12.1%. Mortality was 4.3% in patients hospitalized in medical wards versus 41.7% in patients transferred to ICU (*p* < 0.001). The majority of patients (74.1%) received levofloxacin as definitive therapy, followed by macrolides (16.4%), and combination of levofloxacin plus a macrolide (9.5%). In the multivariate analysis, diabetes (OR 8.28, CI 95% 2.11–35.52, *p* = 0.002), bilateral pneumonia (OR 10.1, CI 95% 2.74–37.27, *p* = 0.001), and cardiovascular events (OR 10.91, CI 95% 2.83–42.01, *p* = 0.001), were independently associated with ICU admission, while the receipt of macrolides/levofloxacin therapy within 24 h from admission was protective (OR 0.20, CI 95% 0.05–0.73, *p* = 0.01). Patients who received a late anti-*Legionella* antibiotic (> 24 h from admission) underwent urinary antigen test later compared to those who received early active antibiotic therapy (2 [2–4] vs. 1 [1–2] days, *p* < 0.001).

**Conclusions:**

Admission to ICU carries significantly increased mortality in patients with diagnosis of LP. Initial therapy with an antibiotic active against *Legionella* (levofloxacin or macrolides) reduces the probability to be transferred to ICU and should be provided in all cases until *Legionella* etiology is excluded.

## Introduction

*Legionella* spp. is a causative agent in both sporadic and epidemic community-acquired pneumonia (CAP), but also in hospital outbreaks [1]. The introduction of urine antigen testing provides an early diagnosis of *Legionella* pneumonia (LP) in the majority of cases, reducing the risk of misdiagnosis and the delay in the administration of effective antibiotic therapy [2]. However, LP remains an infection associated with substantial morbidity and mortality [3]. Intensive care unit (ICU) admission is recognized as an important risk factor for mortality, but unfortunately few data are reported about predictors of severe pneumonia and ICU admission in patients with confirmed LP [4].

The aim of this study was to identify factors independently associated with ICU admission in a series of consecutive patients affected by LP.

## Materials and methods

This observational study was conducted between 2012 and 2019 on consecutive adult patients enrolled at the University hospital of Pisa, Italy. LP was diagnosed if two or more of the following were present: (1) rales, rhonchi, bronchial breath sounds, fever (> 38.0 °C), tachycardia, chills, dyspnea, coughing, chest pain; (2) presence of new consolidation(s) on chest X-ray; (3) diagnosis of *Legionella* spp. infection, defined by *Legionella pneumophila* serogroup 1 antigen in urine. The study was conducted according to the principles stated in the Declaration of Helsinki. The local Ethical Committee approved the study (approval number 1446).

Data on demographic characteristics, comorbidities, antibiotic, and concomitant therapy were retrospectively collected. Stratification of the severity of pneumonia at presentation was quantified by the Pneumonia Severity Index (PSI) and CURB-65 score [5]. CAP and hospital acquired pneumonia (HAP) were defined according to standard definitions [6]. Sepsis and septic shock were defined according to Sepsis-3 definition [7]. Cardiovascular event (CVE) included: (1) non-ST elevation myocardial infarction (NSTEMI); (2) ST elevation myocardial infarction (STEMI); (3) stroke; (4) a new episode of atrial fibrillation (AF); (5) deep venous thrombosis (DVT) and/or pulmonary embolism (PE); (6) new or worsening heart failure (HF); or (7) cardiovascular death [8].

Patients with pneumonia at the time of diagnosis underwent collection of blood cultures, detection of *Legionella pneumophila* serogroup 1 antigen in urine performed by immunochromatographic method (NOW Legionella Urinary Antigen Test; Binax Inc., Portland, ME), and culture of respiratory specimens.

To identify risk factors associated with the primary endpoint (ICU admission), univariate and multivariate analyses were performed. To detect significant differences between groups, we used the chi square test or Fisher's exact test for categorical variables and the two-tailed *t* test or Mann–Whitney test for continuous variables, when appropriate. Continuous variables were reported as mean ± standard deviation or median and interquartile ranges (IQRs) according to distribution; numbers and percentages were reported for categorical variables. Comparison of demographics, comorbidities and baseline variables (recorded at pneumonia onset) between patients who needed admission to ICU and those who did not was performed. Time from admission to receipt of anti-*Legionella* antibiotic (levofloxacin or macrolides) was dichotomized in two categories: <  = 24 h and > 24 h from admission. All variables statistically significant at univariate analysis (*p* < 0.05) were included in the multivariate analysis to identify predictors of ICU admission. Multivariate analysis using logistic regression prediction models was constructed using a forward stepwise procedure; 95% confidence intervals (CI) and odds ratios (OR) were calculated. Statistical significance was established at 0.05. All reported p values are two-tailed.

The results obtained were analyzed using commercially available statistical software packages (SPSS, version 20.0; SPSS Inc, Chicago, Illinois).

## Results

During the study period, 116 patients with LP were identified. Overall, 33 (28.4%) underwent invasive or non-invasive mechanical ventilation, and 24 (20.7%) were admitted to ICU. Thirty-day mortality was 12.1%. An acute CVE was diagnosed in 24 (20.7%) patients (12 new episodes of AF, 4 new or worsening HF, 4 DVT/PE, 2 NSTEMI, 1 STEMI and 1 cardiovascular death). The rate of ICU admission among patients who developed CVEs was 50%.

As reported in Table 1, the 30-day mortality was 4.3% in patients admitted to medical wards compared to 41.7% in patients admitted to ICU (*p* < 0.001). Patients who needed ICU admission received an early (within 24 h from admission) antibiotic therapy including a drug covering *Legionella* less frequently compared to those who did not (54.2 vs. 80.4%, *p* = 0.008).

**Table 1:** Univariate analysis of predictors of ICU admission in the study population

Variables	No ICU admission *N* = 92 (%)	ICU admission *N* = 24 (%)	*p*
Baseline characteristics			
Age, mean ± SD	71.2 ± 14.2	65.7 ± 14.7	0.97
Male sex	59 (64.1)	17 (70.8)	0.634
CAP	67 (72.8)	17 (70.8)	0.804
HAP	5 (5.4)	3 (12.5)	0.359
Charlson comorbidity index, mean ± SD	3.3 ± 2.1	3.1 ± 1.9	0.78
Chronic heart disease	12 (13)	3 (12.5)	1.0
Chronic liver disease	3 (3.3)	3 (12.5)	0.102
Diabetes	12 (13)	10 (41.7)	**0.003**
Neoplasm	17 (18.5)	3 (12.5)	0.762
Chronic renal failure	9 (9.8)	4 (16.7)	0.465
Hemodialysis	0	2 (8.3)	**0.041**
COPD	15 (16.3)	6 (25)	0.374
Immunocompromised status	22 (23.9)	6 (25)	1.0
Clinical features and disease severity			
Delirium at pneumonia onset	36 (39.1)	12 (50)	0.36
Respiratory rate ≥ 30 breaths/min	5 (5.4)	8 (33.3)	**0.001**
Heart rate ≥ 125 beats/min	13 (14.1)	11 (45.8)	**0.002**
Hypotension (MAP < 65 mmHg)	8 (8.7)	6 (25)	**0.04**
Fever (body temperature > 37.5 °C)	79 (85.8)	20 (83.3)	0.741
PSI class, mean ± SD	3.6 ± 1	4.1 ± 0.8	**0.025**
CURB-65, mean ± SD	1.3 ± 0.8	1.8 ± 1	**0.027**
Septic shock	1 (1.1)	9 (37.5)	** < 0.001**
Radiological and laboratory findings			
Pleural effusion	33 (35.9)	16 (66.7)	**0.01**
Bilateral pneumonia	23 (25)	16 (66.7)	** < 0.001**
Serum sodium < 130 mmol/L	12 (13)	7 (29.2)	**0.038**
Leukocytosis (leukocytes ≥ 10.000/µL)	54 (58.7)	14 (58.3)	1.0
Leukopenia (leukocytes < 4.000/µL)	7 (7.6)	2 (8.3)	1.0
Hyperglycemia at pneumonia onset (> 11 mmol/L)	4 (4.3)	5 (20.8)	**0.015**
Lactate > 2 mmol/L	13 (14.1)	16 (66.7)	** < 0.001**
Platelets < 100.000 mm^3^	9 (9.7)	3 (12.5)	0.712
LDH (U/L), mean ± SD	265.8 ± 131.2	465 ± 247	** < 0.001**
PaO2/FiO2 ratio < 250	27 (29.3)	14 (58.3)	**0.03**
Antimicrobial therapy			
Levofloxacin therapy	70 (76.1%)	15 (62.5%)	0.180
Macrolide therapy	17 (18.5%)	5 (20.8%)	0.793
Macrolide + levofloxacin therapy	5 (5.4%)	2 (8.3%)	0.595
Macrolide/levofloxacin therapy within 24 h from admission	74 (80.4%)	13 (54.2%)	**0.008**
Outcomes			
Mechanical invasive or non-invasive ventilation	12 (13)	21 (87.5)	** < 0.001**
Cardiovascular events	12 (13)	12 (50)	** < 0.001**
Median length of hospitalization, mean days ± SD	8.3 ± 3.7	26.1 ± 19.2	** < 0.001**
30-day mortality	4 (4.3)	10 (41.7)	** < 0.001**

*ICU* intensive care unit, *SD* standard deviation, *CAP* community-acquired pneumonia, *HCAP* healthcare-associated pneumonia, *HAP* hospital-acquired pneumonia, *COPD* chronic obstructive pulmonary disease, *HIV* human immunodeficiency virus, *LDH* lactic acid dehydrogenase, *PSI* pneumonia severity index, *ARDS* acute respiratory distress syndrome

Bold values indicate statistical significance (*p* < 0.05)

Overall, the median time from diagnosis of pneumonia to *Legionella* urinary test was 2 (IQRs 1–3) days. Patients admitted to ICU underwent *Legionella* urinary test later than those admitted to medical wards (2 [1–3] days versus 1 day [1–2], *p* = 0.042). Patients treated with an anti-*Legionella* antibiotic after 24 h from admission underwent urinary antigen test later than those who received active antibiotic therapy within 24 h (2 [2–4] vs. 1 [1–2] days, *p* < 0.001).

At the multivariate analysis (Table 2), diabetes (OR 8.28, CI 95% 2.11–35.52, *p* = 0.002), bilateral pneumonia (OR 10.1, CI 95% 2.74–37.27, *p* = 0.001), and cardiovascular events (OR 10.91, CI 95% 2.83–42.01, *p* = 0.001), were independently associated with ICU admission, while receipt of macrolides/levofloxacin therapy within 24 h from admission was protective (OR 0.20, CI 95% 0.05–0.73, *p* = 0.01).

**Table 2:** Multivariate analysis of predictors of ICU admission in the study population

Variables	OR	95% CI	*p*
Cardiovascular events	10.91	2.83–42.01	0.001
Bilateral pneumonia	10.1	2.74–37.27	0.001
Diabetes	8.28	2.11–35.52	0.002
Macrolides/levofloxacin therapy within 24 h from admission	0.20	0.05–0.73	0.01

*ICU* intensive care unit, *OR* odds ratio, *CI* Confidence intervals

## Discussion

Our data showed that some conditions such as diabetes, development of acute CVEs, and a bilateral involvement at chest radiograph are major predictors of ICU transfer. Furthermore, the administration of an initial (within 24 h from admission) antibiotic therapy covering *Legionella* is associated with reduced risk of ICU transfer. Thus, our study suggests that the initial antibiotic therapy of CAP should ever include a drug covering *Legionella* spp. until this etiology is excluded by microbiological tests.

A significant proportion of patients with LP require mechanical ventilation and admission to ICU (20.7% and 28.4% of our patients, respectively). Thus, it is important to identify predictors of ICU admission when the patients arrive at the Emergency Department. Remarkably, we found a strong association with diabetes and development of CVEs. Diabetes is a recognized risk factor for early and late mortality in patients with CAP [9], and is a condition frequently associated with the development of acute CVEs [10, 11]). Most CVEs occur in patients with underlying cardiovascular disease [8]. These data suggest that among patients with severe LP a careful evaluation of cardiovascular parameters should be performed at admission and during the hospital stay: among patients with risk factors for CVE, such as diabetic ones, Troponin and BNP levels should be measured and a strict surveillance, with the use also of cardiovascular ultrasound, is needed to recognize and to treat acute CVEs that may be fatal for the patient.

On the other hand, we found that the administration of an early antibiotic regimen containing at least one drug active against *Legionella* (macrolides or levofloxacin) was a factor significantly associated with reduced risk of ICU admission. We also noted that urinary antigen test for *Legionella* was not performed at hospital arrival in all cases, but after a median of 2 days. A delay in the administration of antibiotics active against *Legionella* was then directly correlated to a delay in the performance of urinary antigen test. These findings lead to some important considerations: first, it is mandatory to perform urinary antigen test for *Legionella* at the time of hospital admission in all cases of severe CAP; this is a recommendation already contained in the current guidelines for CAP [12]. Second, if the test is not available at the time of CAP diagnosis, initial antimicrobial therapy should include a drug with in vitro activity against *Legionella* (azithromycin, clarithromycin, or levofloxacin); to this end, a recent systematic review found no difference in the effectiveness of fluoroquinolones vs. macrolides in reducing mortality among patients with LP [13], although very few data compared the two classes of antibiotics in some settings such as immunosuppressed patients, severe patients needing ICU admission and patients with nosocomial legionellosis [14]. Finally, a positive or negative urinary antigen test prompts withdrawal or continuation of antibiotic treatment directed at *Legionella* pathogen. This strategy improves the antimicrobial prescriptions, by reducing the number of unnecessary antibiotics.

Of importance, no serious adverse events (AEs) directly related to levofloxacin/macrolides administration were recorded in our study population. However, in patients with preexisting cardiovascular diseases an evaluation of the possible AEs should be carefully performed before starting therapy [15].

Our study has several limitations. First, the retrospective design of the study is an intrinsic limitation; second, the relative small sample size does not permit to obtain definite conclusions. Anyway, considering that *Legionella* is an uncommon etiology of pneumonia, our series is one of the largest published in the last years; finally, as regard to decisions adopted in patients at the end of life, in particular “do not resuscitate order” and preclusion to invasive ventilation, there was not a pre-defined protocol and the decision was taken by the physician in charge according to the individual decision and conditions of each patient. It is also important to underline that the urinary antigen test (that includes only *Legionella* serogroup 1) has low sensitivity: a recent paper reported that, compared to PCR molecular test, urinary test results appeared false negative in the 44.4% of cases of LP and a total of 39.4% (26/66) diagnosis probably would have been missed or delayed without a syndromic approach [16]. Thus, physicians should consider to treat cases with a high probability of LP but negative urinary test [17]. Future prospective studies will clarify this point.

In conclusion, the knowledge of predictors for ICU admission could stimulate future studies to understand what therapeutic approaches should be more useful in critically-ill patients, to reduce unfavorable outcomes like CVEs that are associated with increased risk of ICU admission and progression to death.
